# Special Issue—“Multimorbidity Development and Evolution: Clinical Implications”

**DOI:** 10.3390/jcm10163450

**Published:** 2021-08-04

**Authors:** Alberto Zucchelli, Amaia Calderón-Larrañaga, Davide Liborio Vetrano

**Affiliations:** 1 Department of Information Engineering, University of Brescia, 25121 Brescia, Italy; alberto.zucchelli.1@ki.se; 2 Aging Research Center, Department of Neurobiology, Care Sciences and Society, Karolinska Institutet and Stockholm University, 17165 Stockholm, Sweden; amaia.calderon.larranaga@ki.se

Multimorbidity, the co-existence of multiple chronic diseases in the same individual, is not only extremely common in older persons but is also strongly associated with several poor health outcomes [1,2]. Persons affected by multimorbidity are characterized by high levels of complexity and heterogeneity. Decreased physical and cognitive function, frailty, reduced quality of life, unspecific signs and symptoms, and increased risk of drug-related problems are only some of the features that contribute to the challenges linked to the clinical management of multimorbid patients [3,4,5,6]. Furthermore, evidence-based guidelines and effective models for integrated care targeting multimorbid patients are still lacking.

This Special Issue published in the *Journal of Clinical Medicine* aimed to collect high-quality studies that represent the most recent advancements in multimorbidity research. More specifically, two studies here focused on polypharmacy. Using electronic health record data from more than 900,000 multimorbid patients aged 65+ years, Troncoso-Mariño et al. [7] showed a significant increase in the proportion of medication-related problems with increasing levels of polypharmacy, and an independent association between such problems and mortality over four years of follow up. On the other side, Bosch-Lenders and colleagues [8] conducted a randomized controlled trial involving almost 800 participants prescribed with more than 5 chronic medications to investigate the impact of a stepwise medication review by general practitioners. They found that, compared with usual care, the intervention resulted in a higher proportion of stopped medications and a reduced number of doses, as well as better mental health outcomes.

Another two studies targeted the well-known relationship between multimorbidity and poor health outcomes from new perspectives, employing innovative statistical techniques. Akugizibwe and colleagues [9] showed that different patterns of multimorbidity, obtained using an advanced clustering technique, were strongly associated with being acutely hospitalized over a five-year follow-up. Subjects within the *cardiovascular diseases, anemia, and dementia* pattern were the ones exhibiting the highest risk. Furthermore, Vinjerui et al. [10] explored the way occupational position modifies the association between multimorbidity and mortality using data from more than 30,000 Norwegian adults. Interestingly, compared to women, men had a significantly higher risk of dying when both multimorbidity and low socio-economic position were present.

Three studies focused on patient-centered care. Based on data from more than 4750 persons, Ricci-Cabello et al. [11] investigated the association between self-reported safety outcomes and multimorbidity. Remarkably, this relationship seems to be profoundly modified by sex, leading to new and interesting research questions. The mixed methods systematic review by González-González and colleagues [12] synthesized the results of 22 studies evaluating early clarification of end-of-life care preferences in persons with multimorbidity. They found high levels of heterogeneity and a significant impact of hypothetical health status on patients’ decisions. Garg and colleagues [13] summarized novel strategies to conduct research on multiple chronic conditions in order to develop care pathways and interventions able to benefit persons affected by multimorbidity.

Lastly, in their study entitled “*AI and Big Data in healthcare: towards a more comprehensive research framework for multimorbidity*”, Majnarić and colleagues [14] underlined the need to integrate electronic health data, clinical workflows and national/international research infrastructures in order to exploit novel methodologies supported by artificial intelligence, and to deliver effective care to persons affected by multimorbidity.

In conclusion, the article collection included in this Special Issue of the *Journal of Clinical Medicine* shows how novel methodologies and comprehensive and longitudinal datasets can be exploited to investigate the complexity underlying multimorbidity, opening new fascinating research avenues and highlighting, once again, the pivotal role of patient-centered and personalized care in the management of persons affected by multimorbidity.

## References

[1] Vetrano D.L., Calderon-Larranaga A., Marengoni A., Onder G., Bauer J.M., Cesari M., Ferrucci L., Fratiglioni L. (2018). An International Perspective on Chronic Multimorbidity: Approaching the Elephant in the Room. J. Gerontol. Biol. Sci. Med. Sci..

[2] Zucchelli A., Vetrano D.L., Grande G., Calderon-Larranaga A., Fratiglioni L., Marengoni A., Rizzuto D. (2019). Comparing the prognostic value of geriatric health indicators: A population-based study. BMC Med..

[3] Calderón-Larrañaga A., Vetrano D.L., Ferrucci L., Mercer S.W., Marengoni A., Onder G., Eriksdotter M., Fratiglioni L. (2019). Multimorbidity and functional impairment-bidirectional interplay, synergistic effects and common pathways. J. Intern. Med..

[4] Vetrano D.L., Palmer K., Marengoni A., Marzetti E., Lattanzio F., Roller-Wirnsberger R., Lopez Samaniego L., Rodriguez-Manas L., Bernabei R., Onder G. (2019). Frailty and Multimorbidity: A Systematic Review and Meta-analysis. J. Gerontol. Biol. Sci. Med. Sci..

[5] Marengoni A., Zucchelli A., Vetrano D.L., Armellini A., Botteri E., Nicosia F., Romanelli G., Beindorf E.A., Giansiracusa P., Garrafa E. (2021). Beyond Chronological Age: Frailty and Multimorbidity Predict In-Hospital Mortality in Patients With Coronavirus Disease 2019. J. Gerontol. Biol. Sci. Med. Sci..

[6] Aloisi G., Marengoni A., Morandi A., Zucchelli A., Cherubini A., Mossello E., Bo M., Di Santo S.G., Mazzone A., Trabucchi M. (2019). Drug Prescription and Delirium in Older Inpatients: Results From the Nationwide Multicenter Italian Delirium Day 2015–2016. J. Clin. Psychiatry.

[7] Troncoso-Mariño A., Roso-Llorach A., López-Jiménez T., Villen N., Amado-Guirado E., Fernández-Bertolin S., Carrasco-Ribelles L.A., Borras J.M., Violán C. (2021). Medication-Related Problems in Older People with Multimorbidity in Catalonia: A Real-World Data Study with 5 Years’ Follow-Up. J. Clin. Med..

[8] Bosch-Lenders D., Jansen J., Stoffers H.E.J.H.J., Winkens B., Aretz K., Twellaar M., Schols J.M.G.A., van der Kuy P.-H.M., Knottnerus J.A., van den Akker M. (2021). The Effect of a Comprehensive, Interdisciplinary Medication Review on Quality of Life and Medication Use in Community Dwelling Older People with Polypharmacy. J. Clin. Med..

[9] Akugizibwe R., Calderón-Larrañaga A., Roso-Llorach A., Onder G., Marengoni A., Zucchelli A., Rizzuto D., Vetrano D.L. (2020). Multimorbidity Patterns and Unplanned Hospitalisation in a Cohort of Older Adults. J. Clin. Med..

[10] Vinjerui K.H., Bjorngaard J.H., Krokstad S., Douglas K.A., Sund E.R. (2020). Socioeconomic Position, Multimorbidity and Mortality in a Population Cohort: The HUNT Study. J. Clin. Med..

[11] Ricci-Cabello I., Yañez-Juan A.M., Fiol-deRoque M.A., Leiva A., Llobera Canaves J., Parmentier F.B.R., Valderas J.M. (2021). Assessing the Impact of Multi-Morbidity and Related Constructs on Patient Reported Safety in Primary Care: Generalized Structural Equation Modelling of Observational Data. J. Clin. Med..

[12] González-González A.I., Schmucker C., Nothacker J., Nury E., Dinh T.S., Brueckle M.-S., Blom J.W., van den Akker M., Röttger K., Wegwarth O. (2020). End-of-Life Care Preferences of Older Patients with Multimorbidity: A Mixed Methods Systematic Review. J. Clin. Med..

[13] Garg T., Polenick C.A., Schoenborn N., Jih J., Hajduk A., Wei M.Y., Hughes J. (2021). Innovative Strategies to Facilitate Patient-Centered Research in Multiple Chronic Conditions. J. Clin. Med..

[14] Majnarić L.T., Babič F., O’Sullivan S., Holzinger A. (2021). AI and Big Data in Healthcare: Towards a More Comprehensive Research Framework for Multimorbidity. J. Clin. Med..

